# Proteomic analysis of the EhV-86 virion

**DOI:** 10.1186/1477-5956-6-11

**Published:** 2008-03-17

**Authors:** Michael J Allen, Julie A Howard, Kathryn S Lilley, William H Wilson

**Affiliations:** 1 Plymouth Marine Laboratory, Prospect Place, The Hoe, Plymouth, PL1 3DH, UK; 2 Cambridge Centre for Proteomics, Department of Biochemistry, University of Cambridge, Tennis Court Road, Cambridge, CB2 1QR, UK; 3 Bigelow Laboratory for Ocean Sciences, 180 McKown Point, PO Box 475, W. Boothbay Harbor, Maine, 04575-0475, USA

## Abstract

**Background:**

*Emiliania huxleyi *virus 86 (EhV-86) is the type species of the genus *Coccolithovirus *within the family *Phycodnaviridae*. The fully sequenced 407,339 bp genome is predicted to encode 473 protein coding sequences (CDSs) and is the largest *Phycodnaviridae *sequenced to date. The majority of EhV-86 CDSs exhibit no similarity to proteins in the public databases.

**Results:**

Proteomic analysis by 1-DE and then LC-MS/MS determined that the virion of EhV-86 is composed of at least 28 proteins, 23 of which are predicted to be membrane proteins. Besides the major capsid protein, putative function can be assigned to 4 other components of the virion: two lectin proteins, a thioredoxin and a serine/threonine protein kinase.

**Conclusion:**

This study represents the first steps toward the identification of the protein components that make up the EhV-86 virion. Aside from the major capsid protein, whose function in the virion is well known and defined, the nature of the other proteins suggest roles involved with viral budding, caspase activation, signalling, anti-oxidation, virus adsorption and host range determination.

## Background

*Emiliania huxleyi *is the most numerically abundant coccolithophore in the world's oceans and is well known for forming vast blooms that can cover up to 10,000 km^2 ^[1,2]. Viruses have been shown to be a major cause of *E. huxleyi *bloom termination [3-5].* Emiliania huxleyi *virus 86 (EhV-86) is the type species of the genus *Coccolithovirus *within the family *Phycodnaviridae *[3]. EhV-86 was originally isolated from a coccolithophore bloom off the coast of England in 1999 and is a large, double-stranded DNA (dsDNA) virus that infects the marine coccolithophorid *E. huxleyi *[4]. The fully sequenced 407,339 bp genome is predicted to encode 473 protein coding sequences (CDSs) and is the largest *Phycodnaviridae *sequenced to date [5]. Of the 473 predicted CDSs just 66 are annotated with functional product predictions on the basis of sequence similarity or protein domain matches. With the completion of the EhV-86 genomic DNA sequence and its annotation our research has now focused on the functional analysis of the gene products. Essential to the functional analysis is the identification of the proteins associated with the virion particle. Due to the relative simplicity of virus proteomes, 1-DE followed by LC-MS/MS was selected for proteomic analysis to determine the protein composition of the EhV-86 virion.

## Methods

Viral particles were purified from the lysate of an *E. huxleyi *1516 culture previously infected with EhV-86. Briefly, *E. huxleyi *strain 1516 was cultured in 10 litres of f/2 medium at 15°C in a Sanyo MLR-350 incubator with 16 h: 8 h light-dark illumination [6]. Exponentially growing (10 litres, 1.2 × 10^6 ^cells ml^-1^) cells were infected with 10 ml of fresh EhV-86 lysate as described previously [5]. Once clearing of the host culture was observed (5 days later), the lysate was passed through a 0.2 μm Supor membrane filter (Pall) and the filtrate concentrated by tangential flow filtration (Vivaflow200, Sartorius) to 50 ml. Virus particles were purified by CsCl gradient centrifugation (1.1 g/ml, 1.2 g/ml, 1.3 g/ml, 1.4 g/ml). CsCl-purified virus was dialysed against 30 mM Tris pH 7.4, 1 mM EDTA for 24 h and stored at 4°C. Virion proteins were then precipitated overnight at -20°C in 0.1 M ammonium acetate in methanol. Following centrifugation at 3 000 g for 10 mins, the pellet was washed in 80% 0.1 M ammonium acetate solution then again in 80% acetone. The protein pellet was desiccated to remove traces of acetone, then run on a 10% linear gradient 1-D SDS PAGE mini-gel and stained with colloidal coomassie.

The next stage was the identification of proteins within the gel. Approximately 1 mm slices were cut successively from the whole length of each track, 16 in total. The whole track was excised in this way in order to identify as many proteins as possible, and not just those which stained strongly with colloidal coomassie. Proteins within the gel pieces were first reduced, carboxyamidomethylated, and then digested to peptides using trypsin on a MassPrepStation (Waters, Manchester, UK). The resulting peptides were applied to a LC-MS/MS. For LC-MS/MS, the reverse phase liquid chromatographic separation of peptides was achieved with a PepMap C18 reverse phase, 75 μm i.d., 15-cm column (LC Packings) on a nanoAcquity LC system (Waters) attached to QTOF Premier (Waters) mass spectrometer. The MS/MS fragmentation data achieved was used to search the National Center for Biotechnology Information database using the MASCOT search engine [7]. Probability-based MASCOT scores were used to evaluate identifications. Only matches with P < 0.05 for random occurrence were considered significant. The data has been submitted to the PRIDE database under accession number 3182 [8]. Functional and structural annotation was predicted using InterProScan [9,10]. Similarity searches were performed using BLASTP against nonredundant protein sequences [11,12], and transmembrane domains were predicted using HMMTOP v2 [13].

## Results and Discussion

**Figure 1:**
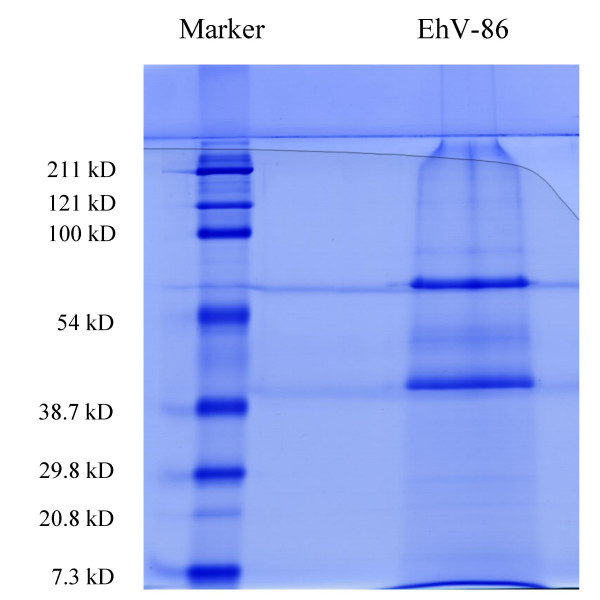
SDS PAGE of EhV-86 virion proteins.

An indeterminate number of faint protein gel bands were visible by SDS-PAGE in the 10 to 200 kDa range, which were dominated by two major bands at 60 kDa and 40 kDa (Figure 1). LC-MS analysis revealed that the virion of EhV-86 is composed of at least 28 proteins (Tables 1 and 2). The 60 kDa band seen by SDS-PAGE (Figure 1) is likely to correspond to the major capsid protein (predicted weight of 59.9 kDa), the 40 kDa band is likely to be a composite of the protein products from ehv067, ehv100, ehv149, and ehv175 with predicted weights of 41.9, 40.0, 40.0, 40.6 kDa, respectively (Tables 1 and 2).

**Table 1:** Proteins identified by LC-MS in purified EhV-86 virions.

TREMBL	Gene	Expression Profile^a^	Number of peptides	MW^b ^(kDa)	Mascot^c^
Q4A3B2	ehv015	2–4 h p.i.	1	14.6	79
Q4A399	ehv034	> 4 h p.i.	2	18.7	117
Q4A398	ehv035	1–2 h p.i.	16	141.4	600
Q4A397	ehv036	2–4 h p.i.	2	18.6	97
Q4A395	ehv038	2–4 h p.i.	1	12.5	48
Q4A378	ehv055	unknown	3	34.1	59
Q4A373	ehv060	> 4 h p.i.	1	212.2	207
Q4A366	ehv067	> 4 h p.i.	4	41.9	145
Q4A348	ehv085	2–4 h p.i.	22	59.9	1224
Q4A333	ehv100	2–4 h p.i.	2	40.0	208
Q4A2Y5	ehv149	1–2 h p.i.	8	40.0	285
Q4A2W6	ehv168	> 4 h p.i.	4	18.6	239
Q4A2V9	ehv175	> 4 h p.i.	2	40.6	59
Q4A2V2	ehv182	> 4 h p.i.	4	22.7	248
Q4A2U4	ehv189	unknown	2	45.4	109
Q4A2U2	ehv191	1–2 h p.i.	1	93.8	46
Q4A2T8	ehv195	> 4 h p.i.	1	22.1	57
Q4A2T3	ehv200	> 4 h p.i.	3	34.1	157
Q4A2N2	ehv250	> 4 h p.i.	1	11.9	100
Q4A2H9	ehv301	> 4 h p.i.	2	32.0	139
Q4A2F4	ehv325	> 4 h p.i.	1	15.8	43
Q4A2E6	ehv333	Unknown	1	13.5	35
Q4A2D9	ehv340	> 4 h p.i.	1	14.4	48
Q4A2C3	ehv356	Unknown	2	81.0	39
Q4A2C1	ehv358	> 4 h p.i.	1	17.2	45
Q4A237	ehv442	> 4 h p.i.	1	19.4	58
Q4A225	ehv454	2–4 h p.i.	3	74.4	119
Q4A218	ehv461	1–2 h p.i.	4	32.9	36

^a ^Data from Allen *et al*., 2006; p.i. indicates the time post infection when the transcript is first seen [25]. ^b^Predicted MW. ^c^The highest score is shown in the case of protein identification in multiple bands.

**Table 2:** Unique peptides used in the identification of proteins from the EhV-86 virion.

**Gene**	**Peptides used for identification of protein product**
ehv015	RDIILDPNASPSDKR
ehv034	KCIAPDYNKN, KVLNETVSGYFRR
ehv035	KDRPLISENGRY, KDSEIEDLEEQNNSLDRD, KEGYDQNFIGVPSYAVRD, KGIIGVALLEGKG, KIPYVYLNPYLKR, KITAPTAALAAEAAKL, KLAGVYGCGSKT, KLATTVASDIETRK, KNILSGDLEKE, KNYDDSVFFKD, KQIETITAELEPLAEKD, KQMEQLQFEKD, KTSTDLANCTTKV, KVGGPYTVISRN, RATAQSEHVAQLLSIETNKN, RLSNLGVLSTNNQILNKN,
ehv036	KESEADLAEAKR, RELGEATDDLGDAKK
ehv038	KTTLSDITAEIADKR
ehv055	KDDVDAWKE, KDDVDAWKEESFVMRA, KTDFNSAVVKS
ehv060	KIDSWEPGELAELYVDSTRV
ehv067	KELNLVLPPGTKG, KLAVIEEIDNKL, KLIIPAETARH, RYMTPLDVARE
ehv085	KANKDAGDHFNFSGIGGRD, KDAGDHFNFSGIGGRD KDAGDHFNFSGIGGRDPVVSAELLFNNTARV, KEQLIAEAKN, KFTNGLAGLLYSN-, KIVLPGLKV, KVGGATIDTIWSELLFAMEELMGRA, KYNAAPLPVAAQMQSTEMPDFDYAYWTEAIGFHLIKR, RASLECTYVHLEAAERD, RDALTANAGTQLIVQHQAHLQQVSSNNVTARL, RDPVVSAELLFNNTARV, RLDSVELALTLQDDFGAAHDANSELFVFARS, RLTETIGRT, RNVPISDDHLRA, RQEQILYVPLPWYFTKH, RQGDLLSWMYLKI, RQGDLLSWMYLKI, RRLTETIGRT, RRPTELMKA, RRPTELMKA, RSNLVVLHAERN, RVTQKPAVWWRA
ehv100	KTTPAIGLGPPDKY, KGTCIGNLTQCTTEKG
ehv149	KCIPDLATICTGKL, KKYDCAPGTKV, KLEPGADNNCVIKA, KLNNVSTGAKK, KVGPLGEKC, KYDCAPGTKV, RAAAAWAATRG, RGMAGSAAGATSSAAKS
ehv168	KNSALMEMVKS, KSTMGAGELEVARQ, KWTGAAAAGAAAPSAADVIYKR, RGVYGPQPAGSDSSTGKT
ehv175	RRPPNILVKM, RYFEDIFNNPRN
ehv182	KEISDPEIVDLKY, KSTCMFEADRS, KYDEESSSPARK, RFVVGDFIINNQGKL
ehv189	KVVDSLYDFRI, RYNAQQSIRD
ehv191	KQNLGQSDGNLLRA
ehv195	RSNNQYNVQRR
ehv200	KSNGYDDNFVGVNKS, KVMAVSATGTTARV, RVNVSPYWPRN
ehv250	KSFEDAANTPGYLSARS
ehv301	RSMNPNDIRT, RSNEVNDTMIARS
ehv325	KEQPNTVSGERV
ehv333	KGYDVAAVQRI
ehv340	KAIGEGMEPGMIRA
ehv356	RGQTDPSQNPVVDTRF, KNPSIIGAAEKY
ehv358	KSADELNTLVKE
ehv442	KYANGSNVTLYYDPKN
ehv454	KIPTATVTTRQ, RWSGDYLEIKK, KSAVTSITLLTDLEQVRV
ehv461	KTNAIELRR, KVDVYSLSPKN, RLTEELRF, RVGAHGPVEIRV

Virus particles are essentially composed of structural proteins and nucleic acids. Only one protein identified in this study has a known function associated with it. The role of the major capsid protein is well defined in viral systems and it typically comprises approximately 40% of the total virion protein mass in phycodnaviruses [14]. Major capsid proteins consist of two consecutive "jelly roll" domains (antiparallel β-barrels) and are a conserved component of the capsid structures in ssRNA, dsRNA, ssDNA and dsDNA viruses [14,15].

It is surprising that of the remaining 27 proteins identified in this study, 23 are predicted to be membrane proteins (Table 3). It is possible that these proteins are associated with an internal membrane, akin to that observed in other *Phycodnaviridae *[16,17]. However, electron microscopy imagery and flow cytometry data has shown that virus release occurs via budding at the host membrane (unpublished). This would suggest that virion particles may be coated in a lipid-protein membrane as they are released from infected cells. One or more of the membrane proteins identified here may be responsible for coordinating this viral budding through the formation of lipid rafts at the plasma membrane. This hypothesis is further enhanced by the previous identification of a sphingolipid biosynthesis pathway in the virus genome [5,18]. Sphingolipids are essential structural components of membranes and are found to be enriched in lipid rafts [18]. An enveloped virus release mechanism has been shown to occur in the retroviruses, paramyxoviruses, orthomyxoviruses and filoviruses [19]. Indeed, this type of mechanism has also been shown to occur with poxviruses, a virus family distantly related to the coccolithoviruses. Hitherto, phycodnaviruses were not thought to be associated with an outer membrane, but the identification of these putative membrane proteins in the EhV-86 virion and the budding release mechanism suggests this is a distinct possibility for the coccolithoviruses.

**Table 3:** Analysis of proteins identified in the EhV-86 virion.

	Protein Analysis			
Gene Number	Top Blast Hit^a^	Blast Score^a^	TMs^b^	InterProScan Results^c^

ehv015	hypothetical protein, *Trichomonas vaginalis *G3.	0.009	1	No hits reported.
ehv034	predicted protein, *Ostreococcus lucimarinus*	0.016	1	No hits reported.
ehv035	similar to SMC2 protein, *Bos taurus*	0.058	2	No hits reported.
ehv036	HlyD family secretion protein, *Agrobacterium tumefaciens*	0.004	2	No hits reported.
ehv038	hypothetical protein, *Bos taurus*	0.32	1	No hits reported.
ehv055	hypothetical protein, *Methanothermobacter thermautotrophicus*	5e^-06^	6	No hits reported.
ehv060	No significant match	n/a	1	C type lectin 2 domain
ehv067	Hypothetical protein, *Giardia lamblia*	1.4	0	No hits reported.
ehv085	major capsid protein, Heterosigma akashiwo virus 01	7e^-39^	0	Capsid domain (iridovirus like)
ehv100	predicted protein, *Nematostella vectensis*	5e^-10^	2	No hits reported.
ehv149	hypothetical protein, *Aedes aegypti*	0.43	2	C type lectin 1 domain
ehv168	hypothetical protein, *Novosphingobium aromaticivorans*	2.4	1	No hits reported.
ehv175	Putative serine/threonine protein kinase, *Populus tomentosa*	0.66	0	Protein Kinase
ehv182	diaminopimelate decarboxylase, *Streptococcus pneumoniae*	0.48	1	No hits reported.
ehv189	pol-like protein, *Nasonia vitripennis*	2.0	0	No hits reported.
ehv191	No significant match	n/a	1	Proline rich extensin signature
ehv195	hypothetical protein, *Salinispora arenicola*	0.27	2	No hits reported.
ehv200	hypothetical protein, *Bacillus sp*.	0.23	1	No hits reported.
ehv250	GCN5-related N-acetyltransferase, *Rhodopseudomonas palustris*	9.6	1	No hits reported.
ehv301	NB-ARC domain containing protein, *Oryza sativa*	0.31	0	No hits reported.
ehv325	envelope glycoprotein, Simian immunodeficiency virus	1.1	1	No hits reported.
ehv333	CRISPR-associated protein, Cse1 family, *Pseudomonas mendocina*	0.35	2	No hits reported
ehv340	Putative fimbrial associated sortase-like protein, *Corynebacterium diphtheriae*	0.42	1	No hits reported.
ehv356	No match	n/a	1	No hits reported
ehv358	hypothetical protein, *Paramecium tetraurelia*	2e^-08^	1	Thioredoxin domain
ehv442	conserved hypothetical protein, *Stigmatella aurantiaca*	0.008	2	No hits reported.
ehv454	hemocyanin isoform 1, *Nucula nucleus*	2.2	2	No hits reported.
ehv461	Fatty acid/phospholipid synthesis protein, *Herminiimonas arsenicoxydans*	2.6	1	No hits reported

^a^BLASTP analysis [12] against nonredundant protein sequences performed on 12^th ^December 2007. ^b^Transmembrane (TM) domains predicted by HMMTOP v2.0. ^c^Functional and structure predicted by InterProScan [10].

Despite intensive database interrogation, no putative function can be assigned to 23 of the 28 proteins identified in this study (Table 3). Besides the major capsid protein, putative function can be assigned to 4 proteins: two lectin proteins, a thioredoxin and a serine/threonine protein kinase. Lectin proteins are well known for their specific and reversible binding to carbohydrate moieties and may function as a binding site during virus adsorption to the host cell. The presence of a putative thioredoxin protein is intriguing since their role as antioxidants is well documented [20], particularly since EhV-86 virions have been shown to be sensitive to reactive oxygen species [21]. Indeed, elevated levels of cellular reactive oxygen species have been shown to be present in the latter stage of infection, suggesting the role of the thioredoxin protein may be to prevent damage during virion assembly [22]. The protein kinase is likely to be involved in an as yet unidentified signalling pathway.

No known components of transcriptional machinery, DNA repair or modification were detected in this study, despite being encoded on the viral genome. Several factors may account for the lack of data for these proteins including the relative abundance, size and hydrophobicity of the proteins. Other related viruses from the Nucleocytoplasmic Large DNA Virus (NCLDV) family such as the mimivirus and Vaccinia virus virions have been shown to contain 114 and 75 proteins respectively, so it is likely that, further and more extensive characterisation will reveal a larger number of proteins in the EhV-86 virion [23-25].

Previous work has shown that 39 virus gene transcripts can be detected 1 hour into infection, a further 194 two hours post infection, and a further 71 at 4 hours post infection [25]. Interestingly, 14 of the 28 proteins identified here fail to be expressed even at 4 hours post infection (see Table 1, genes expressed > 4 h post infection), suggesting that the majority of virion construction and assembly occurs later than 4 hours post infection. This would make sense if they are packaged into the capsid, since their genes are probably expressed last.

Intriguingly, six proteins (EHV301, EHV325, EHV333, EHV340, EHV356 and EHV358) have been detected in the virion whose genes are located in a 100 kbp region of unknown origin or function [26,27]. Many of the genes in this region (but none of these six) are associated with a novel promoter element and are the only genes expressed during the first hour of infection [25]. Transcriptional work has shown that the novel promoter associated genes are among the most highly expressed during infection, however not one of their gene products has been identified in the virion proteome in this study [5,26]. Of particular interest is the presence of a caspase cleavage site (YVAD) in EHV325. Host caspase activity (induced by coccolithoviruses upon infection) has recently been postulated to play a fundamental role in the viral replication strategy [28]. The cleavage of EHV325 by an activated host caspase-like protease, prior to its incorporation as a mature protein into the virion, may account for delay in virus infection observed when caspase function is inhibited in *E. huxleyi*. Caspases are responsible for the initiation and execution of programmed cell death (PCD), a process which is also thought to be affected by ceramide [28,29]. Since the virus genome contains gene homologues for an almost complete biosynthetic pathway for ceramide production, it is clear PCD may have some important role to play during infection. It is speculative to suggest that EHV325, in its newly identified role as a virion component, may be involved in this process.

There are 4 proteins identified whose genes are located at a distinct loci of the EhV-86 genome: EHV034, EHV035, EHV036 and EHV038. Although no known function has been determined for these proteins, their presence in the virion particle is intriguing since a microarray based genome wide survey has suggested that all four genes are either absent or their sequence is sufficiently divergent to cause a negative hybridisation in two Norwegian coccolithovirus strains [30]. Furthermore, ehv034 and ehv301 were found to be absent (or their sequence sufficiently divergent) in nine and seven of the twelve strains tested, respectively. The presence, absence or divergence of the products of these genes in virions may account for the variability in host range observed for different coccolithovirus strains.

## Conclusion

This study represents the first steps toward the identification of the protein components that make up the EhV-86 virion. Hitherto, only one virus protein (the major capsid protein) has been confirmed as part of the virion structure. Here, we have used a simple approach to determine that the EhV-86 virion is composed of at least 28 different proteins. Aside from the major capsid protein, whose function in the virion is well known and defined, the nature of the other proteins suggest roles involved with viral budding, the caspase pathway, signalling, anti-oxidation, virus adsorption and host range determination.

## Competing interests

The author(s) declare that they have no competing interests.

## Authors' contributions

MJA conceived of the study, designed the study, carried out the virus purification and protein preparation, analysed the data and drafted the manuscript. JAH carried out the SDS-PAGE and LC/MS, and analysed the data. KSL helped draft the manuscript. WHW conceived of the study, participated in its design and helped to draft the manuscript. All authors read and approved the final manuscript.
